# Supplementary material for: Differential inclusion of NEB exons 143 and 144 provides insight into NEB-related myopathy variant interpretation and disease manifestation
Source: medRxiv. 2024 Mar 26:2024.03.25.24304535. Preprint. [Version 1] doi: 10.1101/2024.03.25.24304535 (PMC10996755; doi:10.1101/2024.03.25.24304535)

Supplemental Table 1: Pathogenic variants affecting exon 144 in the literature.

Mutation	Second Hit	Clinical phenotype	confirmed Nemaline rods?	Citation
Pathogenic variants affecting exon 144 (NM_001271208)				
ex144: c.21506C>A; p.Ser7169*	Homozygous	Unusual pattern of selective weakness, dystrophic biopsy. Difficulties in waking from anesthesia	YES	Lehtokari et al 2015
ex144; c.21423del; p.Lys7141fs	int112; c.17737-2A>T; p.?	"Unspecified form of NM"	NO	Lehtokari et al 2015
int144: c.21522+3A > G	no second hit identified	Presentation at 9yo with toe walking, no cardiac abnormalities, no other data available for this patient	YES	Lee et al 2017
int144: c.21522+3A > G	Exon 81; c.12148G>T; p.E4050Ter	Childhoodh onset nemaline myopathy	YES	Wan et al 2019
int144: c.21522+119C>G	Homozygous	Presented at 15 months with delayed walking	YES	Laflamme et al 2021

		followed by proximal > distal muscle weakness. Facial/Bulbar weakness noted as well		
Pathogenic variants affecting exon 144 but called exon 143 by NM_001164508.1				
in143:c.21417+3 A>G	ex 133 c.20360_20361insA; p.Thr6787fs	"typical congenital"	YES	wang et al 2020
in143:c.21417+3 A>G	ex120; c.21793C>T; p.Arg5552*	"typical congenital"	YES	wang et al 2020
in143:c.21417+3 A>G	ex45; c.5574_5575ins;p.Lys1859_Lys1860delins	"typical congenital"	YES	wang et al 2020
in143:c.21417+3 A>G	ex18; c.1623delT; p.Asp542Ilefs*15	"typical congenital"	YES	wang et al 2020
in143:c.21417+3 A>G	ex124; c.19211delT; P.Leu6404Argfs*9	"childhood onset"	YES	wang et al 2020
in143:c.21417+3 A>G	ex109; c.17367G>A; p.Trp5789*	"childhood onset"	YES	wang et al 2020
in143:c.21417+3 A>G	In80; c.12019-10G>A;	"typical congenital"	YES	wang et al 2020
in143:c.21417+3 A>G	ex121; c.18917G>A; p.Trp6306*	"childhood onset"	YES	wang et al 2020
in143:c.21417+3 A>G	ex61; c.8479C>T; p.Gln2827*	"childhood onset"	YES	wang et al 2020
in143:c.21417+3 A>G	ex15; c.1263dupA; p.Tyr422fs	"Adult onset"	YES	wang et al 2020
in143:c.21417+3 A>G	ex105; c.16465A>G; p.Lys5489Glu	"Adult onset"	YES	wang et al 2020
in143:c.21417+3 A>G	ex53; c.7212T>G; p.Tyr2404*	"Adult onset"	YES	wang et al 2020
in143:c.21417+3 A>G	ex115; c.18187C>T; p.Arg6063*	"typical congenital"	YES	wang et al 2020
in143:c.21417+3 A>G	ex49; c.6195dupG; p.Tyr2066Valfs*4	"childhood onset"	YES	wang et al 2020

in143:c.21417+3 A>G	ex61; c.8394T>G; p.Tyr2798*	"childhood onset"	YES	wang et al 2020
in143:c.21417+3 A>G	ex3; c.36G>T; p.Glu12Asp	"childhood onset"	YES	wang et al 2020
in143:c.21417+3 A>G	ex150; c.22037A>T; p.Asn7381Ile	"childhood onset"	YES	wang et al 2020
in143:c.21417+3 A>G	ex128; c.19751T>G; p.Met6584Arg	"typical congenital"	YES	wang et al 2020

Supplemental Table 2: Literature on distribution of exon143/144 mRNA

Muscle	Donner et al 2006 MOUSE (143:144) qPCR	Donner et al 2004 HUMAN semi quantitative PCR	Laitila et al 2012 HUMAN Microarray and RT-PCR	Lam et al 2018 HUMAN qPCR	Uapinyoying et al 2020 MOUSE Long read sequencing
**Gastrocnemius Medial**	D0: 1:0 ;D19 4:1; D21 1:1; W6 1:5	Adult 144 only			
**Gastrocnemius Lateral**	D0: 1:0 ;D19 4:1; D21 1:1; W6 1:5	Adult 144 only			
**Tibialis anterior**	D1 1:0; D21 1:1; W6 0:1	Adult expressed both	statistically higher 143 than FDL, TP, FHL		
**Extensor digitorum longus**	D0 2:1; D19 1:1; W6 0:1		Both - no relative expression data		100% 144
**Soleus**	D4 1:1; D19 7:1; D23 4:1; W6 6:5				63% exon 144; 37% exon 143
**Vastus lateralis**	D23 1:1; W4 1:2; W6 1:14		weak exon 143 expression, good exon 144	fast fibers: 143; slow fibers: 144 and 143	
**vastus intermedius**	D23 1:1; W4 1:2; W6 1:14			fast fibers: 143; slow fibers: 144 and 143	
**vastus medialis**	D23 1:1; W4 1:2; W6 1:14			fast fibers: 143;	

				slow fibers: 144 and 143	
**Rectus femoris**	D23 1:1; W4 1:2; W6 1:14	Adult 144 only	Both are expressed, less 144 than adductor longus	fast fibers: 143; slow fibers: 144 and 143	
**Tibialis Posterior**			Less 143 than in the tibialis anterior, expresses both exons		
**Sartorius**			Both expressed, no relative expression data		
**Gracilis**			statistically lower 144 than adductor longus, expresses both		
**Adductor longus**			statistically higher 144 than gracilis, semimembranosus, biceps femoris, rectus femoris, and semitendinosus		
**Adductor magnus**			Both - no relative expression data		
**Semitendinosus**			statistically higher 143 than VL (both transcripts expressed)		
**Semimembranosus**			Expresses both transcripts, less 144 than AL but unclear if more 144 than 143		
**Biceps femoris**			statistically higher 143 than VL, FDL		
**Masseter**	D0 1:1; D14 0:1; W6 0:1				
**Diaphragm**	D0 1:2; D7 1:7; D21/W6 0:1				
**Cardiac**	developmentally expressed equally	Adult 144 only			
**Longus capitis**	exclusively 143				

Supplemental Figure 1

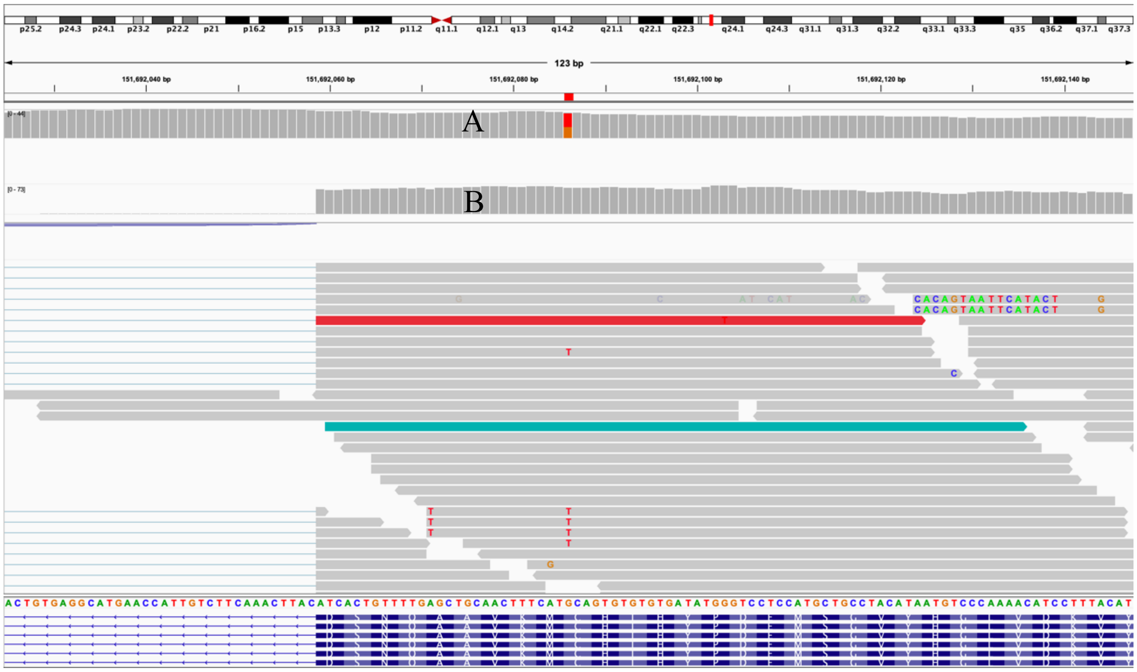

Supplemental Figure 2

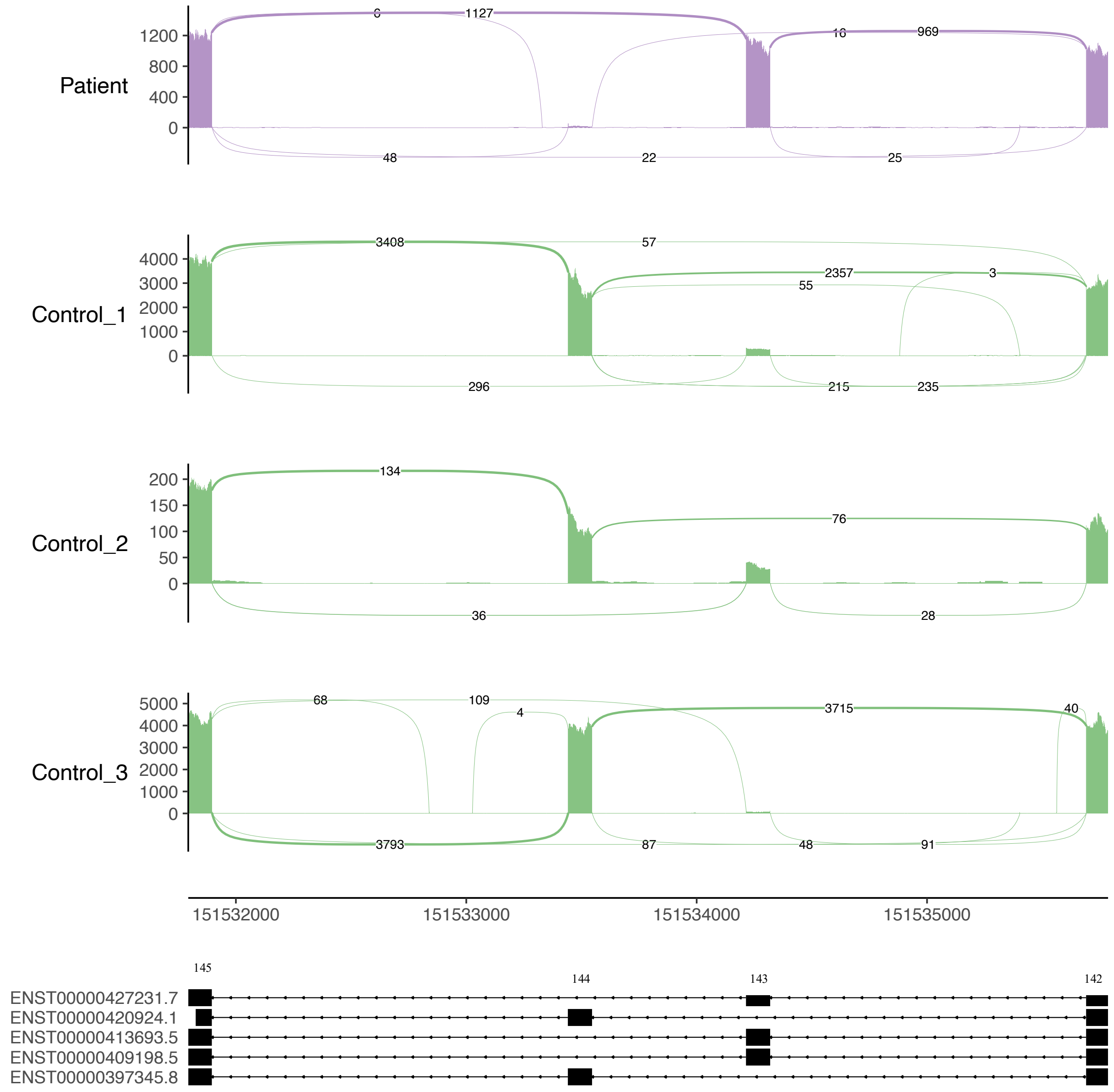

Supplemental Figure 3

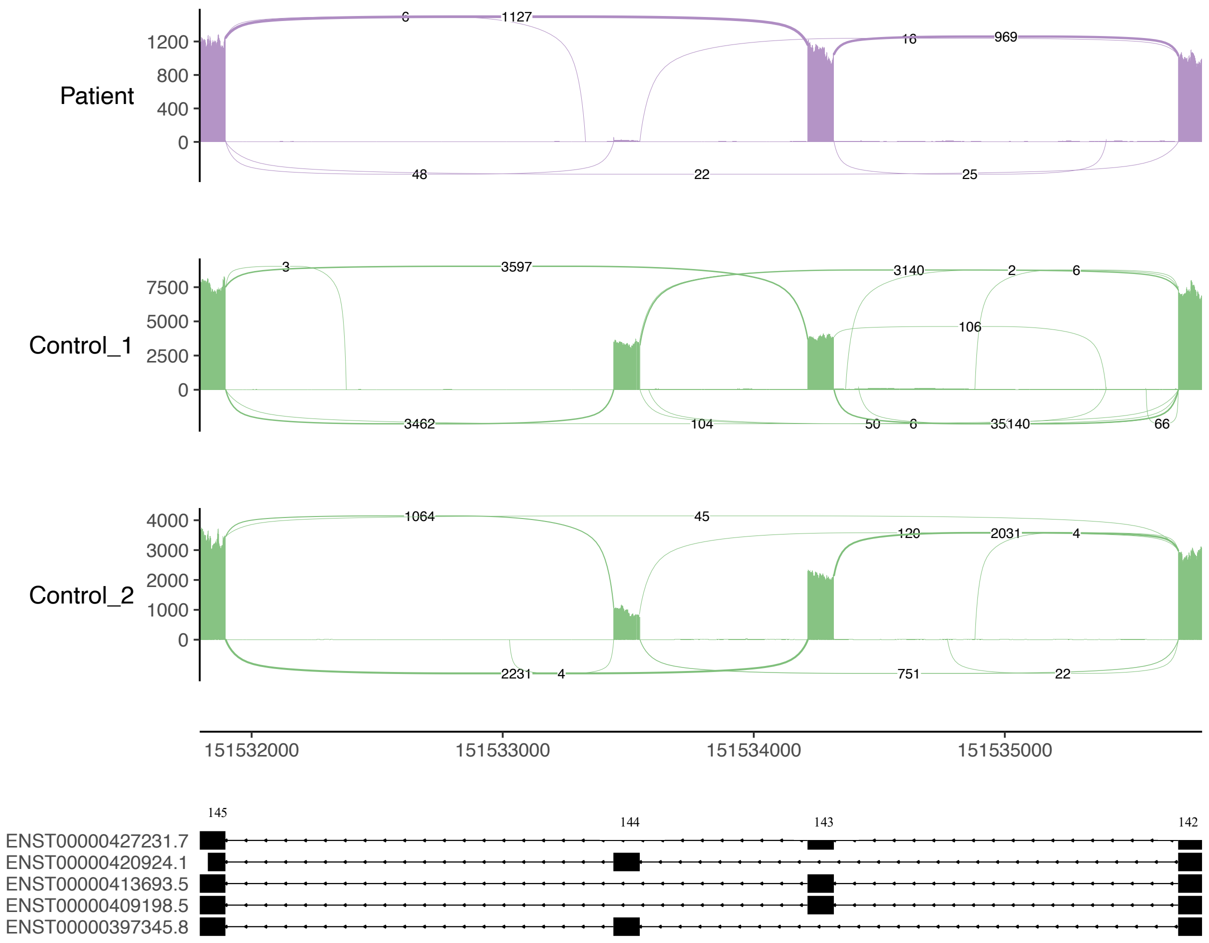

Supplemental Figure 4

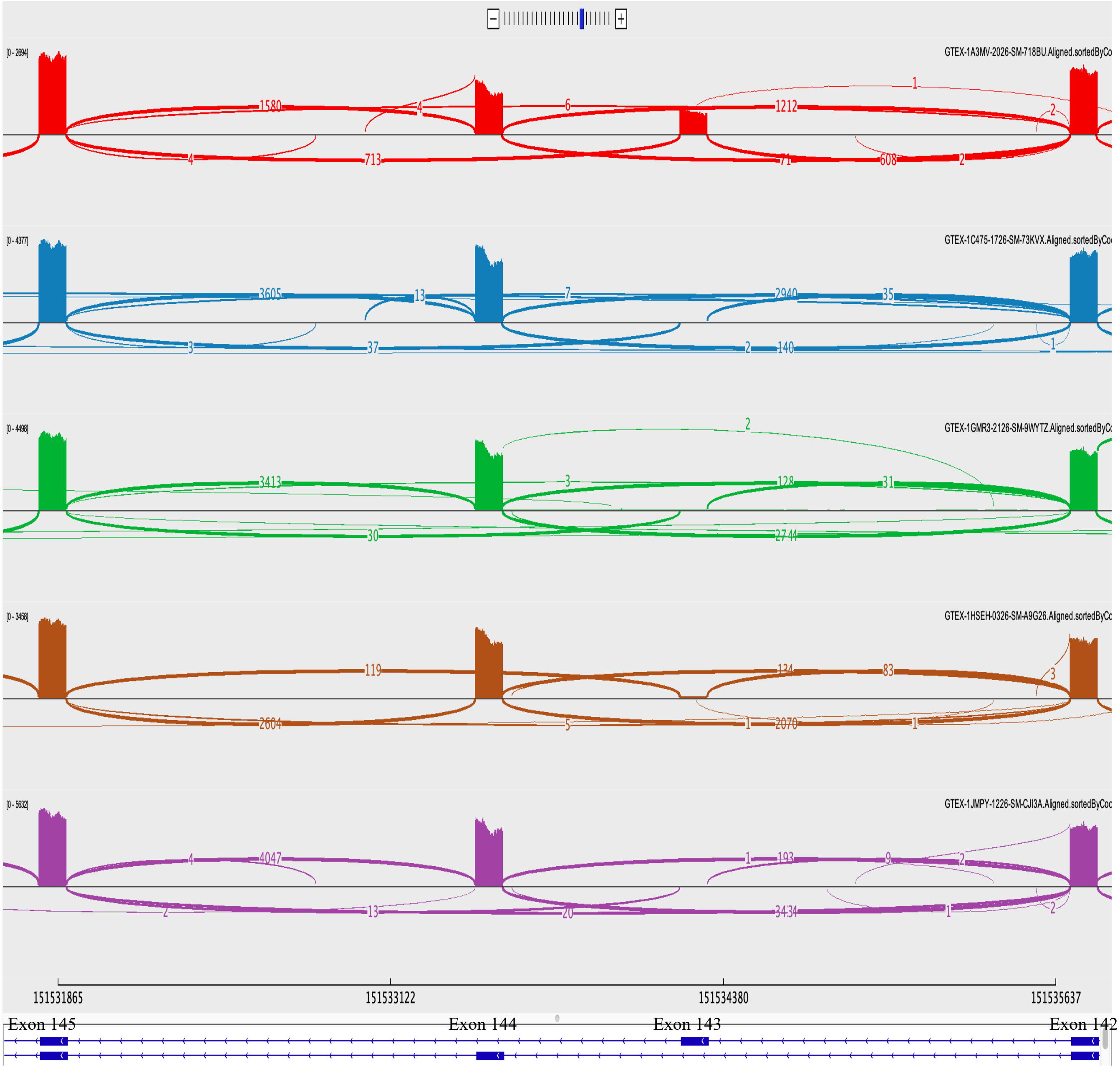

Supplement: Supplement 1 — Supplemental Figure 1: A) Reads aligned to Exon 22 in DNA show heterozygous SNP c.2079C>A; p.(Cys693Ter) present in 50% of reads. B) The variant c.2079C>A; p.(Cys693Ter) is present only in 11 out 64 reads in in RNAseq of P1,consistent with nonsense mediated decay prediction for this truncating variant Supplemental Figure 2: analysis of all isoforms (no junction reads removed) existing in patient and controls between exons 142–145. The only difference between patient and controls is the lack of exon 144 inclusion. Note the 6 splice junction reads in patient starting in intron 144 and ending at exon 145. Patient in purple and three biceps controls in green, all adult (30–50yrs) biceps have a higher read count of exon 144 compared with 143 in adult biceps Supplemental Figure 3: Patient compared with two age and sex matched controls, but not biopsied from the same muscle group. Both exons 143 and 144 are expressed almost equally in control samples, while the patient sample expresses exon 144 only. Again, Note the 6 splice junction reads in patient starting in intron 144 and ending at exon 145, which are not seen in controls. Supplemental Figure 4: five randomly selected GTEX muscle samples biopsied from “below the patella” or “gastrocnemius” all show multiple rare isoforms, including isoforms splicing exon 142 directly to exon 145. Notably, there is no isoform that matches the six reads demonstrating intron 144 extension in our patient. There is variability in exon 143 expression between individual samples, although the lack of precise biopsy knowledge makes drawing conclusions regarding the relative abundance of 143 and 144 from GTEX data difficult. [file media-1.pdf]
